# Is configurational entropy the main stabilizing term in rock-salt Mg_0.2_Co_0.2_Ni_0.2_Cu_0.2_Zn_0.2_O high entropy oxide?

**DOI:** 10.1038/s41467-022-30674-0

**Published:** 2022-05-27

**Authors:** Martina Fracchia, Mauro Coduri, Maela Manzoli, Paolo Ghigna, Umberto Anselmi Tamburini

**Affiliations:** 1 Department of Chemistry, University of Pavia, V.le Taramelli, 12, Pavia, Italy; 2 INSTM, National Inter-University Consortium for Materials Science and Technology, Via G. Giusti 9, 50121 Florence, Italy; 3 Department of Drug Science and Technology and NIS - Centre for Nanostructured Interfaces and Surfaces, Via P. Giuria 9, 10125 Turin, Italy

**Keywords:** Ceramics, Materials chemistry, Thermodynamics

**arising from** Rost et al. *Nature Communications* 10.1038/ncomms9485 (2015)

In their paper “Entropy-Stabilized Oxides,” Rost et al. reported the synthesis of a new Mg_0.2_Co_0.2_Ni_0.2_Cu_0.2_Zn_0.2_O phase (E1) with rock-salt structure as a test case for the concept of high entropy in oxides. Their claim is that “entropy predominates the thermodynamic landscape and drives a reversible solid-state transformation between a multiphase and single-phase state.” Here we use the same thermodynamic considerations by Rost et al. and replicate their experiments but reducing appropriately the configurational entropy. We demonstrate that configurational entropy does not dominate the thermodynamic stability of E1.

We show that most of the experimental evidence supporting the entropic stabilization of E1 applies even when the configurational term ${S}_{{Config}}$ is considerably reduced. We note that the five cations are not equivalent: Mg, Co and Ni form rock-salt oxides, whereas CuO and ZnO exhibit different crystal structures. The first cations are mutually soluble in the whole compositional range. Conversely, CuO and ZnO present limited solubility in rock-salt oxides. Hence, when modifying the stoichiometry to adjust ${S}_{{Config}}$, particular care must be taken to keep constant the stoichiometric ratio of CuO and ZnO, to avoid modification in the phase composition produced by the solubility equilibria.

To demonstrate the relevance of these considerations, we synthesized various solid solutions (SSs) with different numbers of cations, under the constraint that CuO and ZnO molar fractions are equal to 0.2 as in the E1 phase. With reference to figure 2f and 2g by Rost et al.^1^, showing minima in the formation temperatures for the equimolar composition, we note that our approach is different. In fact, to prove the existence of the minima, Rost et al. needed to vary the molar fractions of each component, making difficult a direct comparison between the results.

Figure 1a, b reports the results for binary Ni_0.8_Cu_0.2_O, three-cation Ni_0.6_Cu_0.2_Zn_0.2_O and four-cation Ni_0.4_Co_0.2_Cu_0.2_Zn_0.2_O, compared to E1. The syntheses were performed by solid-state reaction in air at 1000 °C from stoichiometric mixtures of the parent oxides (Aldrich, >99.9%) for 6 days, with intermediate grinding and final quenching to room temperature (RT). All the SSs were obtained as single-phase rock-salt with homogeneous cation distribution down to the nanometer scale.

**Fig. 1:**
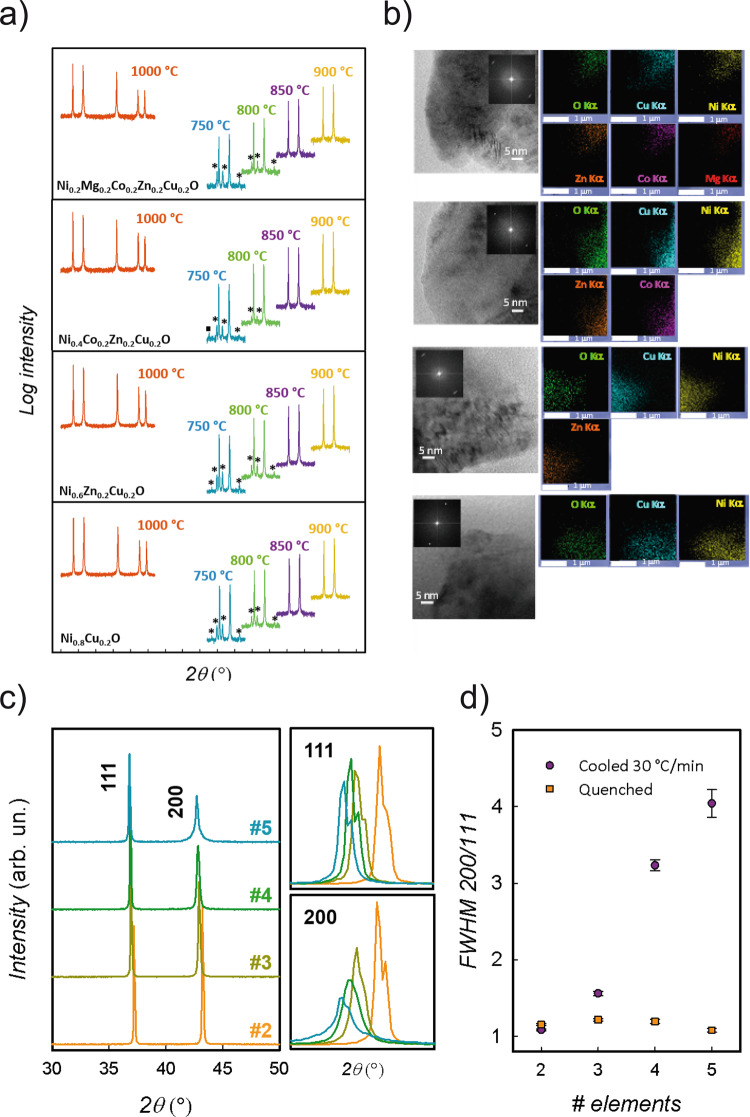
Diffraction and TEM data for all the solid solutions. **a** Powder diffraction patterns for the five-, four-, three- and two-component solid solutions quenched from 1000 °C to RT (red lines). The solid solutions were heated to 900, 850, 800 and 750 °C, and quenched to RT. The corresponding powder diffraction patterns are shown as yellow, violet, green and cyan lines, respectively. Asterisks and squares mark the diffraction peaks of CuO tenorite and spinel phases, respectively. **b** High-resolution transmission electron microscopy (TEM) with corresponding fast Fourier Transform (FFT) and energy-dispersive spectroscopy (EDS) maps for all the elements present. Instrumental magnification: 400 000×. **c** Powder diffraction patterns for the five- (cyan), four- (green), three- (dark yellow) and two- (orange) component solid solutions rapidly cooled (30 °C/min) from 1000 °C to RT. The 111 and 200 reflections are magnified to illustrate better the different broadenings. This is demonstrated in **d**, where the ratio of the full widths at half maximum (FWHM) for the two reflections are plotted as a function of the number of components for the quenched (orange squares) and rapidly cooled (violet circles) samples. Error bars represent confidence intervals.

This result is in contrast with the conclusions by Rost et al., who suggested that only the composition E1 can produce a homogeneous SS down to 875 °C.

This contradiction must be discussed following the same thermodynamic argument by Rost et al.^1^. For 1 mole of E1 SS containing 0.2 moles of CuO and ZnO, the destabilizing term is $0.2\triangle {G}_{{ZnO}}^{{wurtzite}{\to }{HEO}}+0.2\triangle {G}_{{CuO}}^{{tenorite}{\to }{HEO}}=9.4\;{{{{{\rm{kJ}}}}}}{{{{{{\rm{mol}}}}}}}^{-1}$, the transition free energies of CuO and ZnO from their regular tenorite and wurtzite forms to rock-salt being:^2, 3^

1 $$\triangle {G}_{{CuO}}^{{tenorite}{{\to }}{halite}}=25\;{{{{{\rm{kJ}}}}}}{{{{{{\rm{mol}}}}}}}^{-1}$$

2 $$\triangle {G}_{{ZnO}}^{{wurtzite}{{\to }}{halite}}=22\;{{{{{\rm{kJ}}}}}}{{{{{{\rm{mol}}}}}}}^{-1}$$

The configurational entropy is given by ${S}_{{Config}}=-R\mathop{\sum }\nolimits_{i=1}^{n}{{\chi }}_{i}{ln}{{\chi }}_{i}$, where ${{\chi }}_{i}$ is the molar fraction of the *i*-th component; the values for all compositions are reported in Table 1. At the synthesis temperature, i.e., 1000 °C, *TS*_Config_ nearly doubled the value required to transform these oxides into rock-salt. This implies that, at 1000 °C, E1 composition is stable as a single-phase SS, and the three- and four-component compounds with ${{\chi }}_{{CuO}}$ and ${{\chi }}_{{ZnO}}$ = 0.2 should be stable as well. Even binary Ni_0.8_Cu_0.2_O at 1000 °C has *TS*_Config_ = 5.3 kJmol^−1^_,_ which is greater than $0.2\triangle {G}_{{CuO}}^{{tenorite}{\to }{HEO}}$ = 5.0 kJmol^−1^. In fact, all these SSs do form at this temperature.

**Table 1:** Thermodynamic properties of the solid solutions reported in this work.

*Ν*	χ_CuO_	χ_2_	χ_3_	χ_4_	χ_5_	*S*_Config_	*TS*_Config_ (kJ mol^−1^) at 1000 °C	*TS*_Config_ (kJ mol^−1^) at 850 °C	*TS*_Config_ (kJ mol^−1^) at 800 °C	$\triangle {G}_{{CuO},{ZnO}}^{{phase\; transition}}$ (kJ mol^−1^)
2	0.2	0.8				0.5 *R*	5.3	4.7	4.5	5.0
3	0.2	0.2	0.6			0.95 *R*	10.1	8.9	8.5	9.4
4	0.2	0.2	0.2	0.4		1.33 *R*	14.1	12.4	11.9	9.4
5	0.2	0.2	0.2	0.2	0.2	1.61 *R*	17.1	15.1	14.4	9.4

*χ*_i_ is the molar fraction of the i-th component, *N* is the total number of components, and *S*_Config_ is the corresponding configurational entropy. Values of the product *TS*_Config_ are given at some selected temperatures and the overall $\triangle {G}_{{CuO},{ZnO}}^{{phase\; transition}}$ of the structural transitions from tenorite and wurtzite to rock-salt is reported in the last column.

To test the stability at lower temperatures, the SSs were annealed at 750, 800, 850 and 900 °C for 2 h and then quenched to room temperature(Fig. 1a). All compositions showed segregation of tenorite CuO at *T* < 850 °C, while for *T* ≥ 850 °C single phase was retained. The *TS*_Config_ terms at 800 and 850 °C for all compositions are reported in Table 1. At both temperatures, for the two- and three-component oxides, these terms were lower than $\triangle {G}_{{CuO},{ZnO}}^{{phase\; transition}}$, while for the four- and five-component oxides, they were higher than $\triangle {G}_{{CuO},{ZnO}}^{{phase\; transition}}$. Thus, based on configurational entropy only, SSs with two or three cations should not exist at 800 and 850 °C, while they should exist with four or five cations. The presence of an additional impurity phase with the spinel structure (probably Co_3_O_4_), found for the 4-component system at 750 °C, is irrelevant for the above discussion as it disappears at *T* = 800 °C. It is therefore concluded that the stability of these SSs cannot be discussed only in term of ${S}_{{Config}}$, and that additional terms must contribute.

Let us now consider the role of solubility equilibria, starting from the simplest case, the binary Ni_0.8_Cu_0.2_O. The equilibrium phase diagram for this system shows that, at 1000 °C, this composition corresponds to a stable SS. The solubility of CuO in NiO drops rapidly with temperature^4^. However, Fig. 1a, b show that a homogeneous SS in a metastable form was obtained at RT upon quenching. When this SS was annealed at 750 °C, CuO segregated. Further heating at 1000 °C restored the SS. This reversible behavior, similar to that reported by Rost et al. for E1 composition, can be easily explained as a reversible transition between monophasic and biphasic regions of the phase diagram. The solubility limit for this composition is indeed around 800 °C^4^. We suggest that a similar argument can explain the behavior of the other compositions, although the details of the phase equilibria in multicomponent systems are largely unknown. It is known, however, that all binaries within the MgCoNiCuZn/O system exhibit reciprocal solubility above 20% at 1000 °C. A stable rock-salt SS for ${{\chi }}_{{CuO}}$ and ${{\chi }}_{{NiO}}$ < 0.2 at 1000 °C in the system CuO-MgO-NiO was also reported^5^.

Further indications on the stability of the rock-salt SSs were obtained by cooling all the compositions from 1000 °C to RT at 30 °C/min. This rate was fast enough to inhibit CuO segregation, but slow enough to allow structural relaxations. The diffraction patterns at the end of the cooling procedure are shown in Fig. 1c. E1 shows a considerable broadening of all the reflections, except for the 111 family, which is consistent with a tetragonal distortion of the rock-salt structure^6, 7^. The broadening decreases significantly by decreasing the number of components (Fig. 1d), and therefore ${S}_{{Config}}$: thus, decreasing ${S}_{{Config}}$ decreases the tendency of the rock-salt structure to distort from the perfect cubic symmetry. This is a clear indication that the ${S}_{{Config}}$ does not play the simple role of stabilizing the cubic rock-salt structure.

In summary, we have shown that the synthesis of homogeneous rock-salt SSs is possible in the MgCoNiCuZn/O system with two, three or four components, provided that the molar fractions of CuO and ZnO are kept below a limiting value close to 0.2, which is dictated by the high-temperature solubility equilibria. These SSs behave in a quasi-identical way to E1 when quenched at RT and then annealed at intermediate temperatures. Also, the tendency of the rock-salt structure to distort from the cubic symmetry decreases with ${S}_{{Config}}$. All this evidence points toward the fact that, although the contribution of ${S}_{{Config}}$ is undoubtedly present and significant, its role towards the stability of Mg_0.2_Co_0.2_Ni_0.2_Cu_0.2_Zn_0.2_O is limited. ${S}_{{Config}}$ is surely a robust and fruitful approach for controlling the stability of complex oxides, but its role must be carefully analyzed in view of the solubility equilibria under consideration.

## Data Availability

The data that support the findings of this study are available from the corresponding author upon request.
